# Comparison of Two Methods for Assessing the Maxillary Sinus Volume in Patients with and Without Unilateral Cleft Lip and Palate: A Retrospective Cross-Sectional Study

**DOI:** 10.3390/diagnostics16060865

**Published:** 2026-03-14

**Authors:** Aleksandra Kołodziejska, Wojciech Nazar, Bogna Racka-Pilszak, Anna Wojtaszek-Słomińska

**Affiliations:** 1 Department of Conservative Dentistry, Faculty of Medicine, Medical University of Gdańsk, 80-210 Gdansk, Poland; 2 Laboratory of Experimental and Translational Allergology, Department of Allergology, Faculty of Medicine, Medical University of Gdańsk, Smoluchowskiego 17, 80-214 Gdansk, Poland; 3 Medical University of Gdańsk, Al. Zwycięstwa 42 c, 80-210 Gdansk, Poland

**Keywords:** maxillary sinus, cleft lip, cleft palate, surgery, tomography

## Abstract

**Background/Objectives**: The aim of this study was to compare two methods for maxillary sinus volume measurement, assessing their accuracy. The analysis compared the maxillary sinus volume in patients with unilateral cleft lip and palate (UCLP) and in a non-cleft group, using a manual method and a three-dimensional (3D) semi-automated segmentation method. **Methods**: The research was conducted according to the STROBE guidelines. Sixty patients were included in this study: thirty patients with UCLP were in the research group, and the control group consisted of 30 patients with no craniofacial deformities. Cone-beam computed tomography (CBCT) was analyzed. The manual maxillary sinus volume was calculated based on its approximation to two geometric shapes based on mathematical formulas using linear measurements that were performed on all sinus CBCT scans in the maximum diameter in three planes. The semi-automatic segmentation method using ITK-SNAP 3D-imaging software version 4.2.2 was used to automatically calculate the maxillary sinus volume of the sinuses. The manually calculated volume was compared with the automatically calculated one, and statistical analysis was performed. **Results**: The cleft group presented lower values in both the automatic and manually calculated volumes for both the right (automatic: *p* = 0.49; manual *p* = 0.009) and left (automatic: *p* = 0.46; manual *p* = 0.11) maxillary sinuses than the non-cleft group. The cleft group presented statistically significant higher discrepancies in values between the manual and semi-automatic method than the control group (RMSV *p* = 0.0011; LMSV *p* = 0.033; TMSV *p* = 0.003). **Conclusions**: The manual method may not reveal the exact anatomical topography of the maxillary sinuses. In UCLP patients, the maxillary sinus anatomy may be more complex. Therefore, a semi-automated method may be more advisable to preserve the accuracy of the measurements.

## 1. Introduction

Facial development is initiated early in prenatal life. The maxillary sinus emerges at approximately the tenth week of gestation as an outgrowth of the mucosa lining the primitive ethmoidal infundibulum [1]. Its most dynamic growth occurs during early childhood until the age of three, followed by a gradual growth phase associated with dental eruption, reaching maturity in mid-adolescence around age fifteen [2].

The nasal cavity together with the paranasal sinuses plays a crucial role in conditioning inhaled air and protecting the lower respiratory tract from environmental pollutants, allergens, and pathogens. Additionally, the maxillary sinuses reduce the skull’s bone mass while shaping the facial contours. They enhance vocal resonance and act as a shock-absorbing zone during trauma to the midface [3,4]. Disturbances occurring during embryogenesis may lead to the formation of orofacial clefts, which subsequently alter the growth pattern of the maxillofacial complex [5]. Cleft patients frequently present functional impairments involving the maxillary sinuses [6].

The accurate assessment of sinus anatomy is significant in pathological diagnosis and treatment planning. However, because the sinuses are hidden within the midfacial bones, assessing their volume in routine clinical settings is difficult, requiring the use of radiological methods. Some studies have attempted to determine the volume of the paranasal sinuses through invasive or post-mortem techniques, such as measuring skulls or filling cadaveric sinuses with various substances [7,8]. The introduction of computed tomography (CT) has made it possible to analyze anatomical structures more precisely without invasive interventions. According to Ariji et al., the maxillary sinus measurements obtained by CT scans correspond to the measurements on human skulls [9]. Hence, CT is considered to be a reliable method for evaluation of the maxillary sinus [10]. The subsequent development of cone-beam computed tomography (CBCT) has provided comparable diagnostic accuracy while substantially reducing the radiation exposure [11,12,13]. However, CBCT has lower soft tissue contrast and, therefore, may have segmentation limitations [14,15].

Advancements in various software tools have further enabled detailed volumetric analyses of organs, cavities, and specific regions through a range of segmentation techniques, including manual, semi-automated, and fully automated [16]. However, the maxillary sinus volume assessment in the UCLP group remains difficult because of its more complex anatomy. UCLP patients present variations in nasal morphophysiology, such as turbinate hypertrophy, a deviated septum, and modifications to the nasal floor [17,18]. The maxillary sinuses of cleft patients have irregular contours and are often altered, especially in the anterior region, by the surgical interventions performed in the early life stages [19,20]. The irregularities in the bony contour associated with facial and skeletal deformities may affect the segmentation algorithm.

Moreover, all methods have their limitations. The method based on linear measurements may not fully capture the sinus morphology, as they are based on measurements in specific places that may exclude the surface and volume [20]. The manual method, which is considered to be the “gold standard” in segmentation, is time-consuming and dependent on the researcher’s experience and judgment; hence, its result may differ between researchers [21,22].

On the other hand, the semi-automated method is less time-consuming but still requires specific software. All semi- or fully automated methods are prone to algorithm failures. The image quality is also important, as it may affect the threshold and contrast accuracy [21]. Semi-automated methods may still require supervision by a clinician and extensive manual checks [23].

In recent years, attention has been directed toward the evaluation of the maxillary sinus volume in patients with cleft lip and palate. Studies have reported comparisons between the maxillary sinus volume in cleft and non-cleft groups [24,25]. However, there are few data comparing different methods of maxillary sinus volume measurements in this group of patients. To the best of the authors’ knowledge, no article has compared the maxillary sinus volume manual method and three-dimensional (3D) semi-automated segmentation method in unilateral cleft lip and palate (UCLP) patients and a non-cleft group. The aim of this retrospective study was to assess the maxillary sinus volume measurement utilizing manual and semi-automated methods. The null hypothesis was that the semi-automated method will be equally reliable to the manual one in assessing the maxillary sinus volume in both groups.

## 2. Materials and Methods

The protocol of this retrospective study complied with the principles outlined in the Declaration of Helsinki and was approved by the Ethics Committee of the Medical University of Gdansk, Poland (Reference no. KB/163/2025 on 4 April 2025). Written informed consent was obtained from all participants. The study design adhered to the Strengthening the Reporting of Observational Studies in Epidemiology (STROBE) guidelines [26].

### 2.1. Study and Control Group

A total of 60 patients were included in this study. Thirty patients diagnosed with UCLP were selected for the research group, and 30 healthy non-cleft patients were the control group. All participants were between 9 and 20 years old. In the study group, 24 patients presented left-sided UCLP, and 6 presented right-sided UCLP. The study group included 16 males and 14 females, and the mean age of the UCLP group was 13.4 years. The control group also consisted of 30 people (18 males and 12 females) with a mean age of 11.3 years. No formal sample size calculation was performed; however, all available patients were included. Their number was limited. This study is a retrospective pilot study.

### 2.2. Inclusion and Exclusion Criteria

Patients who possessed a full sufficient medical record (medical chart and CBCT covering the region of interest) were included in this study. Patients who did not meet these criteria were excluded from this study. Patients with craniofacial deformities apart from UCLP were excluded from this study.

### 2.3. Radiographic Data Acquisition

In this retrospective cross-sectional study, radiographic data from the Orthodontic Departments covering the period 2017–2025 were reviewed. Patient qualification was performed from 7 April 2025 to 7 May 2025. Measurements were performed from 7 May to 1 July.

Radiographic data were collected using a Carestream CS9300C scanner (Carestream Dental LLC, Atlanta, GA, USA). The parameters were set at 90 kVp, 4.0 mAs, with a scanning time of 8.0 s, an axial slice thickness of 0.18 mm, a voxel size of 0.30 mm^3^, and a field of view (FOV) of 17 × 13.5 cm. The CBCT data were saved in Digital Imaging and Communications in Medicine (DICOM) format.

#### 2.3.1. Manual Method Analysis

**Figure 1:**
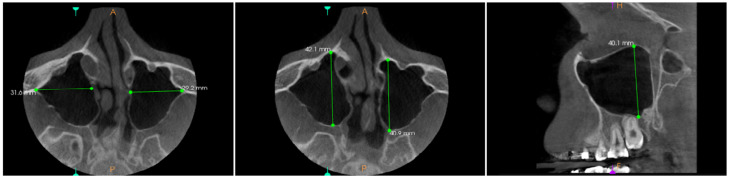
Maxillary sinus linear measurements (axial and sagittal view). CS 3D imaging software, version 3.10.33 (Carestream Dental LLC, Atlanta, GA, USA).

Manual volume calculations were conducted on all sinus CBCT scans according to the method by Przystańska et al. [27]. First, the linear measurements were performed on the maximum diameter in all three planes. The height of the maxillary sinus was measured as the largest distance from the lowest point of the inferior wall to the highest point of the superior wall on the sagittal view. The width of the maxillary sinus was determined as the maximum perpendicular distance between the most prominent points of the medial and lateral walls on the axial view. The length of the maxillary sinus was the longest distance from the most anterior point of the anterior wall to the most posterior point of the posterior wall on the axial view (Figure 1).

Then, the volume of each sinus was calculated based on its approximation to two geometric shapes. These volumes, referred to as manually Maxillary Sinus Volume (mMSV), were determined using the following formulas:

1. V = 4/3 × π × r^3^, representing the sinus as a sphere;
2. V = 1/3 × A × h, representing the sinus as a pyramid.

#### 2.3.2. Semi-Automatic Method Analysis

**Figure 2:**
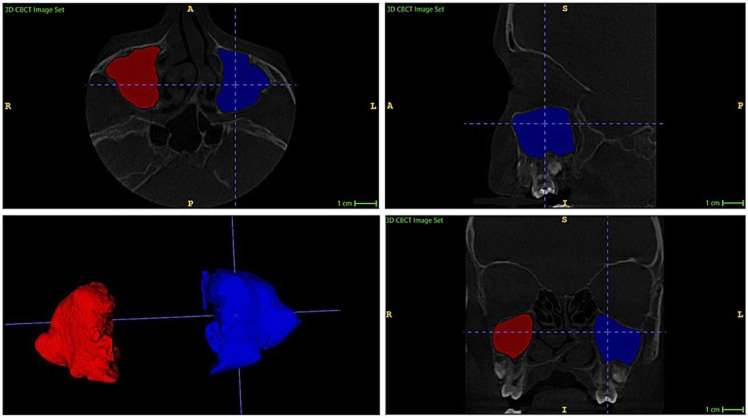
Maxillary sinus volume segmentation with 3D-generated module (coronal, axial, and sagittal view). ITK-SNAP imaging software, version 4.2.2 (Philadelphia, PA, USA).

The automatic Maxillary Sinus Volume (aMSV) volumes of the right and left maxillary sinuses were assessed using ITK-SNAP version 4.2.2 (Philadelphia, PA, USA), which is 3D imaging software. The maxillary sinus segmentation and measurement were based on the bony contours of the sinuses. A semi-automatic segmentation technique was employed to isolate and measure the maxillary sinuses. The surrounding tissues of all paranasal sinuses were removed using the Active Contour (Snake) Segmentation Mode, following an initial pre-segmentation with the Thresholding tool. The software generated a 3D model of each maxillary sinus and calculated its volume in cubic millimeters (Figure 2).

The linear and semi-automated measurements were made twice within a two-week interval by the first author and a month later by the third author. Both raters were trained orthodontists with experience in UCLP cases. The values are the arithmetic mean. In accordance with the ALARA (As Low As Reasonably Achievable) rule, no additional radiographs were taken specifically for this study. All images were obtained as part of ongoing treatment, and all measurements were statistically analyzed.

### 2.4. Logistic Regression

The dependent variables were differences in maxillary sinus volumes calculated using a manual and automated method.

For each parameter, a binary outcome variable was created based on the median value of the respective distribution. Values below the median were coded as 0, and values equal to or above the median were coded as 1. Thus, each model analyzed a dichotomous outcome representing lower versus higher volume difference.

The independent variables included sex, cleft status and age of the patient.

To assess the association between the independent variables and the probability of having a volume difference equal to or above the median (coded as 1), binary logistic regression analysis was performed separately for manual-vs-automated RMSV, LMSV, and TMSV difference variables.

Regression coefficients (β), odds ratios (ORs), 95% confidence intervals (95% CIs), and *p*-values were calculated for each predictor. Odds ratios greater than 1 indicated increased odds of belonging to the group with values equal to or above the median, whereas odds ratios below 1 indicated decreased odds.

### 2.5. Statistical Analysis

Statistical analyses were conducted using the Statistical Package for the Social Sciences (SPSS), version 30.0.0 (SPSS Inc., Chicago, IL, USA), Statistica 10 (StatSoft, Kraków, Poland), and Python 3.10, with the Pandas (v2.1.3) and Scikit-learn (v1.2.1) libraries. A two-tailed *p*-value of less than 0.05 was considered statistically significant. The Shapiro–Wilk test was used to assess the normality of data distribution. Depending on the distribution, group comparisons were carried out using either parametric tests (e.g., Student’s *t*-test, ANOVA) or non-parametric tests (e.g., Mann–Whitney U test, Kruskal–Wallis test). The reliability of the measurements was evaluated by performing intraclass correlation coefficients (ICC).

## 3. Results

The analysis included 60 patients. Twenty-four patients presented left-sided UCLP, with a mean age of 13.4 years, and six patients presented right-sided UCLP, with a mean age of 15.8 years. There were 16 male participants in the study group and 18 in the control group and 14 females in the study group and 12 in the control group. The control group had a mean age of 11.3 years. Detailed descriptive statistics of these outcomes are summarized in Table 1.

The analysis of the groups was conducted, as shown in Table 2. No statistically significant differences were found between the groups in the semi-automatic method for both right (UCLP: 12,240.4 mm^3^ [95% CI: 10,415.6 to 14,065.2] and right non-cleft: 13,037.6 mm^3^ [95% CI: 11,270.8 to 14,804.3]) and left maxillary sinuses (UCLP: 12,094.4 mm^3^ [95% CI: 10,567.9 to 13,620.9] and left non-cleft: 13,102.6 mm^3^ [95% CI:11,340.7 to 14,864.5]). There was statistical significance in the manual method for the right maxillary sinus volume (UCLP: 9502.4 mm^3^ [95% CI: 8226.2 to 10,778.6], with non-cleft: 12,127.2 mm^3^ [95% CI: 10,623.3 to 13,631.1]). The cleft group presented lower values in both the automatic and manually calculated volumes for both the right and left maxillary sinuses than the non-cleft group.

Table 3 presents the discrepancies in the parameters between the two methods. The values presented in the table represent the differences between the volumes calculated using the semi-automated and manual methods, reported separately for the right, left, and total (sum of right and left) maxillary sinus volumes. The volumes are expressed in cubic millimeters and as percentages. According to Table 3, there was a statistically significant difference in all the measurements concerning the variance in the values between the two methods in right and left maxillary sinuses. The cleft group presented higher discrepancies in the values between the manual and semi-automatic methods than the control group. The difference in the total maxillary sinus volume (sum of right and left maxillary sinuses volume) in the cleft vs. non-cleft group (UCLP: 4803.8 mm^3^ [95% CI: 3574.7 to 6032.9]; non-cleft: 2027.1 mm^3^ [95% CI: 668.9 to 3385.4]) was over twice as large, while the difference in percentage was (UCLP: 24.2% [95% CI: 18.8 to 29.6]; non-cleft: 8.8% [95% CI: 3.7 to 13.9]) three times larger for the manually calculated volume. The differences in all the calculated volumes were smaller in the group of patients without craniofacial deformities.

In all the analyses, there were no significant differences between genders.

The analyses of intra- and inter-observer variability revealed consistently high and clinically acceptable reproducibility for all the assessed measurements. The intraclass correlation coefficient (ICC) was higher than 0.90. The intra-observer assessments demonstrated a lower mean bias compared with the inter-observer evaluations. The mean absolute percentage error was 3.5% for intra-observer measurements, whereas the inter-observer error was higher, reaching 6.5%.

### 3.1. Difference in RMSV Volume

In the model assessing differences in RMSV volume, age was the only statistically significant predictor (*p* = 0.02; Table 4). Increasing age was associated with higher odds of RMSV volume difference (OR = 1.39; 95% CI: 1.05–1.83).

Neither sex (OR = 1.43; p = 0.64) nor positive cleft status (OR = 4.91; *p* = 0.073) reached statistical significance. Although cleft status demonstrated a relatively high odds ratio, the wide confidence interval (0.86–27.94) and *p*-value above 0.05 suggest insufficient evidence for an independent association in this model.

### 3.2. Difference in LMSV Volume

Positive cleft status was associated with a six-fold increase in the odds of LMSV volume difference (OR = 6.00; 95% CI: 1.21–29.74; *p* = 0.03). This suggests a strong independent association between the presence of cleft and LMSV differences in automated and manual calculations.

Age was also significantly associated with LMSV volume difference (OR = 1.29; 95% CI: 1.02–1.63; *p* = 0.03), indicating a 29% increase in odds per unit increase in age.

Sex was not significantly associated with LMSV volume difference (OR = 1.80; *p* = 0.38).

### 3.3. Difference in TMSV Volume

In the TMSV model, age again emerged as a statistically significant predictor (OR = 1.31; 95% CI: 1.03–1.68; *p* = 0.03). Each unit increase in age increased the odds of TMSV volume difference by 31%. Neither sex (OR = 1.40; *p* = 0.63) nor cleft status (OR = 2.91; *p* = 0.17) showed statistically significant associations with TMSV volume difference between the automated and manual measurements.

## 4. Discussion

The cleft group in our study presented lower values than the non-cleft group of the right and left maxillary sinuses in both the automatic and manual methods. In the literature, the largest growth in maxillary sinus pneumatization is observed until the end of permanent teeth eruption. Our study group mean age was 11.3 years and 13.4 years, which indicates that the maxillae sinuses were still developing. The study of Jun et al. shows that the maxillary sinus volume was lower in our cleft patient group in relation to the same age of the no-cleft group [28]. Studies conducted by Demirtas et al. focused on the analysis of the maxillary sinus volume in the cleft group, showing similar volumes of maxillary sinuses with our group, and their mean research group age was similar to our study. In Demirtas’ study, the mean age group was 14.9 years, while in our study the mean age was 13.4 years. This study revealed that there is a significant difference between the sides of the maxillary sinus volume in cleft patients [29]. Rodrigues et al. and Yildirim et al. compared the maxillary sinus volume in cleft and non-cleft groups. According to Rodrigues et al.’s study, the maxillary sinus volumes were lower (9728.50 ± 4795.19 mm^3^) in the cleft patient group in comparison with our study group, with their patients aged 4–25. They concluded that the presence of unilateral cleft lip and palate did not affect the maxillary sinus volume [30]. Yildirim et al. compared the maxillary sinus volumes in UCLP, bilateral cleft lip and palate (BCLP), and healthy control groups, showing that the maxillary sinus volumes in the cleft groups were lower than in the healthy control group [31].

There were no statistically significant differences found between the groups in the semi-automatic method for both right and left maxillary sinuses.

In our study, the cleft group presented statistically significantly higher discrepancies in values between the manual and semi-automatic method than the control group. The differences in all the calculated volumes were smaller in the group of patients without craniofacial deformities. These findings of our research highlight the methodological sensitivity of volumetric assessment to craniofacial morphology.

The maxillary sinuses of the UCLP patients often have irregular shapes and are frequently modified due to multiple surgical procedures performed early in life [19,20]. The surgical closure of the cleft impacts further maxillary growth [5]. UCLP patients often present turbinate hypertrophy, deviated septum, and alternations to the nasal floor, possibly because of primary repair surgery, which may impair the growth and function of the maxilla, therefore altering the nasal morphophysiology [32]. The studies of Shetty et al. suggest that the presence of a nasal septal deviation may have a significant effect on the palatal dimensions [33,34]. Therefore, the differences between the methods could be high, where in the non-cleft group the maxillary sinuses often have regular contour and correct anatomy. In addition, regression models support the finding that the presence of clefts may predispose to higher differences between the methods. Moreover, age was consistently a significant positive predictor, whereas sex did not influence any of the measurements. Wide confidence intervals for clefts suggest caution in interpretation, and larger samples may help clarify these effects. Our research suggests that method-specific calibration may be necessary when evaluating anatomically complex cases. Not only is the method’s accuracy itself important but also the patient type may influence the choice of tool used for diagnostic purposes.

In radiographical imaging and anatomical analysis, accurate measurement of structures is critical for diagnosis, treatment planning, and monitoring. The results presented in our study correspond with the findings of Przystańska et al. and Hamdy et al. [27,35]. The sinus volume calculation based on linear measurements is straightforward and widely used in clinical practice. It is relatively fast when measuring simple structures. However, they provide only limited information, as they often reduce complex 3D structures into one or two dimensions, potentially overlooking volumetric or morphological changes [27]. In contrast, automatic 3D segmentation methods may reveal the anatomical complexity of the segmented structure, although they require high-quality imaging data and additional software packages. Their accuracy also depends on algorithm performance and may vary across different imaging modalities or patient populations. According to our findings, the semi-automated method could be more accurate in the assessment of the maxillary sinus volume in patients with clefts. The 3D segmentation method may reveal small recesses in the maxillary sinuses and still remain accurate in the evaluation of the volume of the anatomical structures [36], as the maxillary sinuses in this group of patients may present more complex anatomy. The knowledge of exact maxillary sinus morphology imaging may benefit clinical practice and influence the decisions made by medical practitioners. The precise acquaintance with the recesses of the maxillary sinus may influence the surgeon’s final decision on where to place a dental implant in the UCLP patient and decide whether the patient needs additional surgical procedures such as sinus lift and/or bone augmentation. This knowledge may also influence the orthodontic treatment and the decision on where to place skeletal anchorage in order to achieve the required retention of the miniscrew and support orthodontic movements. The familiarity with the maxillary sinus volume may influence the surgical approach in maxillofacial surgery and otolaryngology especially in endoscopic procedures.

Assessing the accuracy of 3D image segmentation is a crucial step in validating the method used in the study. The goal of segmentation is to separate a 3D image into regions, such as organs, tissues, or cavities. The “gold standard” remains manual segmentation [37], which involves comprehensively labeling the 3D structure in each 2D slice. However, it is a time-intensive task with relatively low inter- and intra-individual reliability and has, therefore, not been widely employed in clinical practice [22]. The evaluation must quantify how closely the predicted segmentation matches the reality. According to Lo Giudice et al., the 3D segmentation tools proved to be accurate and reliable in assessing the volume of the maxillary sinuses and other regions [38]. The study conducted by Weissheimer et al. compared the upper airway volume calculated on the acrylic phantom, which was then assessed by six software programs including ITK-SNAP. The volume of the acrylic oropharynx was known and established as the gold standard. The results showed that the calculation of the software programs was reliable but presented errors. ITK-SNAP revealed less than 2% error in the measurement [39], which in the case of the maxillary sinus volume may not be clinically significant. Evaluation should also consider the quality of segmentation, especially in applications where minor inaccuracies can lead to significant clinical consequences. When dealing with non-regular or small structures, traditional metrics may be biased. In such cases, structure-specific metrics or region-wise analysis may provide a more comprehensive evaluation. The differences in maxillary sinus volume assessment in between methods were not statistically significant. However, the manually calculated sinuses presented lower values. This difference may result from the fact that the manual method estimates the shape and does not include all the anatomical processes of the maxillary sinus structure. ITK-SNAP proved to be a good tool in the assessment of non-regular topography structures [40].

In view of the existing literature, there was no paper that previously compared the manual method based on linear measurement and the semi-automated method of the maxillary sinus volume assessment in UCLP patients. This research demonstrates that cleft anatomy may influence the accuracy and agreement between volumetric assessment techniques. Findings indicate that technique-specific calibration might be required when assessing anatomically complex cases such as UCLP patients opting for a semi-automatic method.

## 5. Limitations and Future Directions

The authors acknowledge a substantial risk of bias in the reported results. This risk primarily arises from the limited sample size and the heterogeneity of the study population, particularly with respect to age. As a retrospective study, the analysis relied on existing medical records, which inherently constrained the cohort selection and resulted in relatively small and heterogeneous groups. Furthermore, the retrospective design precluded the implementation of standardized imaging protocols and uniform data acquisition procedures across all participants.

Although the study groups demonstrated comparable mean ages, considerable interindividual age variability remained. Such variability may influence the anatomical development, morphometric characteristics, and measurement accuracy, thereby introducing additional variability into the results and potentially confounding the observed associations. Age-related differences in tissue maturation and anatomical proportions are especially relevant in studies involving quantitative anatomical assessments and may limit the generalizability of the findings.

Consequently, future investigations should prioritize prospective study designs with larger and more homogeneous cohorts to reduce the selection bias and enhance the statistical power. Standardized imaging and measurement protocols would further improve the data consistency and comparability. In addition, future research may benefit from the application and validation of more advanced artificial intelligence-based methods for volumetric assessment, three-dimensional segmentation, and longitudinal analysis of anatomical development, which could provide more robust and precise insights into structural changes over time.

## 6. Conclusions

In conclusion, this study provides novel evidence that anatomical complexity in UCLP patients not only influences maxillary sinus volume but also affects the agreement between manual and semi-automatic segmentation methods. The manual method may not reveal the exact anatomical topography of the maxillary sinuses. Both methods may be used to calculate the maxillary sinus volume. However, in patients with clefts, whose anatomical structure of the maxillary sinus may be more complex, the semi-automated method may be more advisable to preserve the accuracy of the measurements. Clinically, these findings underscore the importance of selecting segmentation techniques appropriate to the level of anatomical complexity, particularly in cleft populations where precise volumetric evaluation may influence diagnostic accuracy and treatment planning. The accurate maxillary sinus volume measurement and 3D image segmentation of the maxillary sinus anatomy may be helpful for the clinician to more accurately plan orthodontic or surgical treatment. These results contribute to improving the accuracy and reliability of radiographic assessment in patients with craniofacial deformities and enhance precision in the clinical decision-making process during treatment.

## Figures and Tables

**Table 1:** Descriptive statistics of the study group.

	Cleft Group	Non-Cleft Group	*p*-Value
Number of participants	30	30	-
Male	16.0	18.0	0.79
Female	14.0	12.0	0.79
Age	13.4 (11.9–14.9)	11.3 (10.2–12.5)	0.06

Categorical variables are analyzed using counts and percentages. Continuous variables are summarized using medians and corresponding 95% CIs. Paired sample *t*-tests are performed with a significance level of <0.05.

**Table 2:** A comparison of maxillary sinus volumes calculated using two methods.

		Cleft	Non-Cleft	*p*-Value
Automatic maxillary sinus volume	RMSV	12,240.4 (10,415.6–14,065.2)	13,037.6 (11,270.8–14,804.3)	0.49
	LMSV	12,094.4 (10,567.9–13,620.9)	13,102.6 (11,340.7–14,864.5)	0.46
Manual maxillary sinus volume	RMSV	9502.4 (8226.2–10,778.6)	12,127.2 (10,623.3–13,631.1)	0.009 *
	LMSV	10,028.6 (8831.2–11,226.0)	11,985.9 (10,427.3–13,544.5)	0.11

Categorical variables are analyzed using counts and percentages. Continuous variables are summarized using medians and corresponding 95% CIs. * Paired sample *t*-tests are performed with a significance level of <0.05; RMSV: right maxillary sinus volume, LMSV: left maxillary sinus volume.

**Table 3:** Comparative analysis of differences in maxillary sinus volumes.

	Cleft	Non-Cleft	*p*-Value
Difference in RMSV volume [mm^3^ ]	2738.0 (1998.0–3477.9)	910.4 (101.6–1719.2)	0.0011 *
Difference in LMSV volume [mm^3^]	2065.8 (1494.7–2637.0)	1116.8 (436.9–1796.6)	0.033 *
Difference in TMSV volume [mm^3^]	4803.8 (3574.7–6032.9)	2027.1 (668.9–3385.4)	0.003 *
Difference in RMSV volume [%]	27.4 (20.5–34.4)	8.0 (1.9–14.0)	0.00006 *
Difference in LMSV volume [%]	20.9 (15.8–26.0)	9.6 (4.3–14.9)	0.003 *
Difference in TMSV volume [%]	24.2 (18.8–29.6)	8.8 (3.7–13.9)	0.00007 *

Categorical variables are analyzed using counts and percentages. Continuous variables are summarized using medians and corresponding 95% CIs. * Paired sample *t*-tests are performed with a significance level of <0.05; RMSV: right maxillary sinus volume, LMSV: left maxillary sinus volume, TMSV: total maxillary sinus volume.

**Table 4:** Multivariable logistic regression models evaluating predictors of maxillary sinus volumes.

		Coefficient	Odds Ratio	*p*-Value	CI Lower (OR)	CI Upper (OR)
Difference RMSV volume	Sex	0.36	1.43	0.64	0.32	6.34
	Cleft	1.59	4.91	0.073	0.86	27.94
	Age	0.33	1.39	0.02	1.05	1.83
Difference LMSV volume	Sex	0.59	1.80	0.38	0.49	6.68
	Cleft	1.79	6.00	0.03	1.21	29.74
	Age	0.26	1.29	0.03	1.02	1.63
Difference TMSV volume	Sex	0.33	1.40	0.63	0.36	5.43
	Cleft	1.07	2.91	0.17	0.62	13.61
	Age	0.28	1.31	0.03	1.03	1.68

Categorical variables are analyzed using counts and percentages. Continuous variables are summarized using medians and corresponding 95% CIs. Paired sample *t*-tests were performed with a significance level of <0.05; RMSV: right maxillary sinus volume, LMSV: left maxillary sinus volume, TMSV: total maxillary sinus volume.

## Data Availability

The original contributions presented in this study are included in the article. Further inquiries can be directed to the corresponding authors.
